# Heat and Cold-Stressed Individuals of *Pistacia lentiscus* (Mastic Tree) Do Modify Their Secreting Profile

**DOI:** 10.3390/plants11233290

**Published:** 2022-11-29

**Authors:** Aikaterina L. Stefi, Varvara Papaioannou, Theodora Nikou, Maria Halabalaki, Dido Vassilacopoulou, Nikolaos S. Christodoulakis

**Affiliations:** 1 Section of Botany, Department of Biology, Faculty of Sciences, National and Kapodistrian University of Athens, 15701 Athens, Greece; 2 Division of Pharmacognosy and Natural Products Chemistry, Department of Pharmacy, National and Kapodistrian University of Athens, 15771 Athens, Greece; 3 Section of Biochemistry and Molecular Biology, Department of Biology, Faculty of Sciences, National and Kapodistrian University of Athens, 15701 Athens, Greece

**Keywords:** environmental stress, heat and cold stress, Mediterranean plants, secondary metabolites, Dopa decarboxylase, *Pistacia lentiscus*

## Abstract

Seedlings from the germinated seeds of *Pistacia lentiscus* were cultured in plant growth chambers for three months. Then, the plants were separated into three groups. Each group was cultured under different conditions. The first group was left to grow under normal Mediterranean conditions, as those recorded in spring. The other group was subjected to a ten-day heat stress while the last one also suffered a cold stress for ten days. The anatomical features of the leaves (leaf thickness, epidermal cell thickness, number of palisade layers, and development) between these three groups differed. The stressed plants accumulated large amounts of phenolics within their mesophyll cells. The biomass of the cold-stressed plants was minor, while it was high for the control plants. The oxidative stress was hardly detectable in the leaves of the control plants, while their heat-stressed counterparts suffered the highest concentration of reactive oxygen species. Differences concerning the absorption spectra of the three groups of leaves were not significant. An interesting incompatibility between the three groups concerned the expression of L-Dopa Decarboxylase, which climbed significantly in the heat-stressed plants. Finally, an interesting variation was observed concerning the concentrations of some biogenic amines/amino acids. This variation can be correlated to the other stress-induced reactions of the plants and, in some cases, was impressive. In conclusion, environmental stress can shift *Pistacia lentiscus*’ metabolism to synthesize different biogenic products, which can be considered as exploitable for the pharmaceutical or food industry.

## 1. Introduction

Plants thriving in Mediterranean-type ecosystems are considered to be well adapted to the severe heat and drought stress of the summer months and the confined, yet intense, chilling period during the winter months [1]. This adaptation is expressed through various strategies [2,3,4,5,6]. Among them, a major reaction is the biosynthesis of certain secondary metabolites in order to ease the pressure exercised on the primary metabolic pathways, mainly to photosynthesis, by the stressing environmental factors [7,8,9,10,11,12]. Numerous plant secondary metabolites are bioactive compounds with several applications that are used in the food and pharmaceutical industries. Consequently, various plant families, in general, and many plant species, became subjects of scientific interest and are regarded as high-added-value natural sources [13,14,15,16,17,18].

Recently, a certain reaction of Mediterranean plants after severe environmental stress was correlated to the expression of certain secondary metabolites [19,20,21] whose biosynthesis is shared by animals and plants [22,23] and includes the production of animal neurotransmitters, i.e., acetylcholine in the stinging trichomes of the stinging nettle (*Urtica* spp.) as a defense mechanism against herbivory [24,25,26]. Another secondary metabolite synthesized by both plants and animals [12,22,23] is the excessively toxic L-3,4-dihydroxyphenylalanine (L-DOPA) [27]. To clear this toxicity, plant cells rapidly eliminate L-DOPA through an enzymatic conversion catalyzed by L-DOPA DeCarboxylase (DDC). The catecholamine dopamine [26] is the product of this reaction. Dopamine is another well-known mammal neurotransmitter [28] that acts as a precursor of many alkaloids, regulates carbohydrate metabolism, and protects the plants against various pathogens [28].

The mastic tree (*Pistacia lentiscus* L.) is a dioecious, evergreen sclerophyllous shrub, widespread all-round the Mediterranean basin. The secondary shoot of this plant, when injured, secretes an ivory-colored resin. This secreted resin from *P. lentiscus* plants, growing wild or cultivated on the southern part of the Greek island of Chios (east Aegean Sea), has highly appreciated characteristics and a unique, astonishing flavor. This resin, secreted from the trees of Chios, is globally known as mastic or Chios mastic gum. Hippocrates and, later, the Romans, respected this resin as a drug for the prevention of digestive problems and colds. It is referred to as a popular medicine for traditional remedies in *De Materia Medica* [29,30]. Additionally, it was highly prized by the Sultans of the Ottoman Empire, in the middle ages, as a breath freshener and a cosmetic for their harems. Mastic is an expensive plant product with a complex biochemical profile [31] that generates the unique mastic taste. It has antibacterial and antifungal properties [30,32,33] and contains potent antioxidants [34]. It is claimed that mastic can cure peptic ulcers by eliminating the load of *Helicobacter pylori* [35,36], and enhances male sexuality [37]. The curing effect of mastic oil on some types of human cancer has also been reported [38,39]. Therefore, mastic is a famous plant product worldwide, and it is also greatly respected by eastern peoples (Chinese, Japanese).

Considering all the above, and having in mind that Chios is an island with typical Mediterranean climate featuring long, hot, and arid summers as well as cold winter months, imposing plants to severe, timely separated and qualitatively different stresses [1], we decided to conduct an experiment in order to define if qualitative different environmental stresses have any effect on the leaf structure as well as on the function of certain metabolic pathways. We also try to reveal any possible, terminal effect that the stress may have on the biochemical profile of the secreted mastic.

## 2. Results

### 2.1. Microscopy—Leaf Anatomy

**Figure 1:**
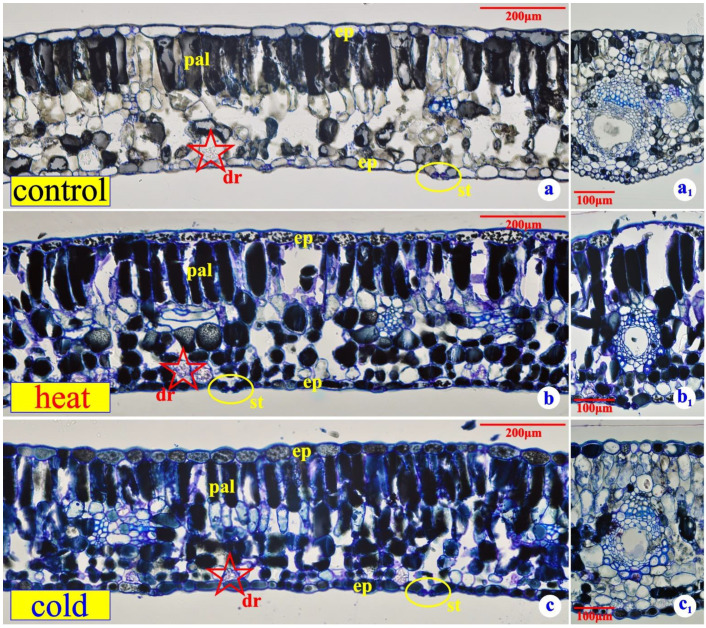
(**a**) Cross section of an epoxy-embedded control leaf. (**a_1_**) Resin ducts from a control leaf. (**b**) Cross section of an epoxy-embedded heat-stressed leaf. (**b_1_**) Resin ducts from a heat-stressed leaf. (**c**) Cross section of an epoxy-embedded cold-stressed leaf. (**c_1_**) Resin ducts from a cold-stressed leaf. ep: epidermis; pal: palisade; dr: druse-type crystal; st: stoma.

Sectioned leaf tissue from epoxy-embedded specimens revealed distinct differences in the leaves of the three groups. Normal leaves appear compact, with one well-defined palisade layer. An inner layer of short palisade-like cells can also be distinguished. The cells of the upper palisade layer accumulate phenolics, many of them in granular form, while the cells of the inner layer are rather free from such inclusions (Figure 1a). The spongy parenchyma is rather loose, with some cells exhibiting druse-type crystals and phenolic inclusions to some extent (Figure 1a). The cells of the epidermal tissue, both adaxial and abaxial, appear flat with no osmiophilic inclusions (Figure 1a). Stomata appear only on the abaxial epidermis (Figure 1a), while the resin ducts seem to retain the normal arrangement of their cells (Figure 1a_1_).

Semithin sections from leaves exposed to the heat stress appear rather different than their control counterparts. The mesophyll is far more compact (Figure 1b), the palisade cells are stuffed with high-density osmiophilic inclusions, intercellular spaces in the spongy parenchyma are limited, and the epidermal cells accumulate phenolics of granular type, while resin ducts seem to be reinforced with elements of the mechanical tissue (Figure 1b_1_). A few idioblasts with druse-type crystals can be observed in the mesophyll, while stomata keep exploiting the lower (abaxial) epidermis.

The leaves exposed to cold stress (Figure 1c) appear rather similar to those exposed to heat stress. All the mesophyll, as well as the epidermal cells, are impregnated with osmiophilic material. Intercellular spaces are missing in the mesophyll. A few idioblasts with druse-type crystals can be observed. Stomata appear on the lower surface only. The resin ducts of the leaves are well protected, encapsulated in mechanical tissue (Figure 1c_1_).

### 2.2. Biomass, Reactive Oxygen Species (ROS), and Pigments

**Figure 2:**
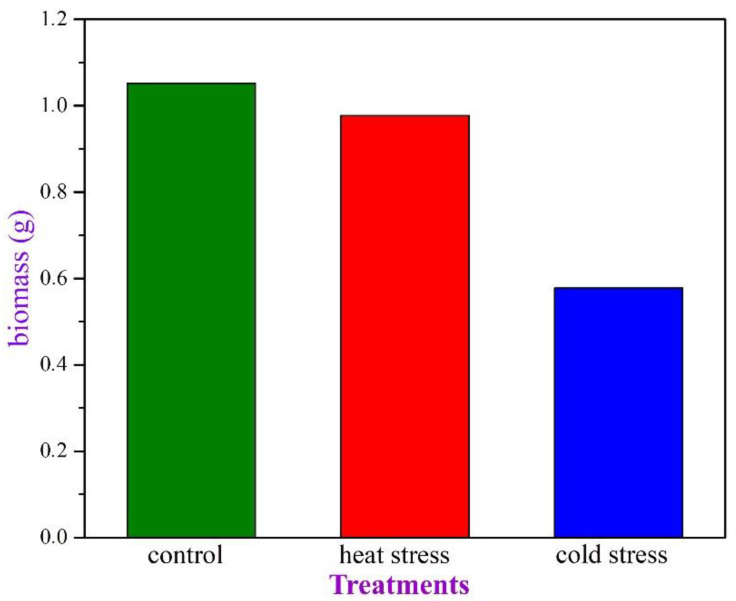
The cumulative biomass—both the above ground parts and the root—for all three groups of plants.

Concerning the total biomass of the plants, in all three groups (Figure 2), we observe that the heat stress does not seem to impede plant growth, while the cold stress causes a remarkable slow-down of plant growth, yielding a total dry weight of about half that of the control plants.

**Figure 3:**
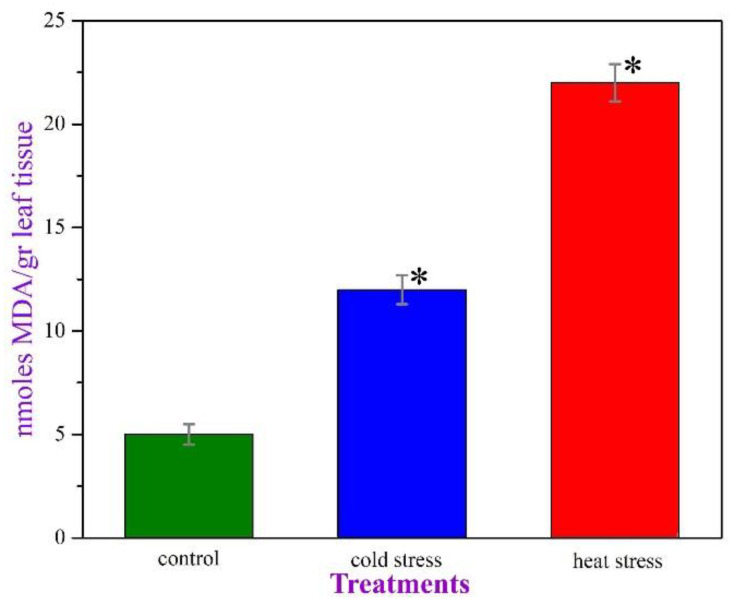
The oxidative stress—ROS—as measured for all three groups of *P. lentiscus* plants. Differences were considered as statistically significant when *p* < 0.05 (*). Error bars represent the standard error of the mean (SEM, *N* = 4, *n* = 1).

The results from the ROS estimation via lipid peroxidation (Figure 3) indicate that the oxidative stress of the plants in the “control” group is negligible, as expected. The cold-stressed plants appear to react, producing an ROS content more than two-fold the quantity recorded for the “control” plants, while the plants exposed to high temperature seem to experience an oxidative stress about twice as severe as that of the cold-stressed plants.

**Figure 4:**
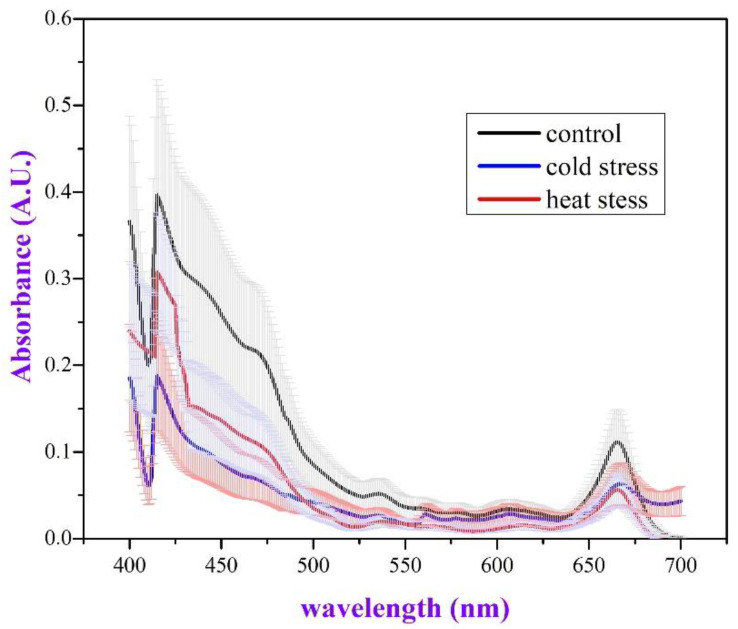
The absorbance spectra of the leaf pigments for all three groups of plants. Error bars represent the standard error of the mean (SEM, *N* = 4, *n* = 1).

The investigation of the absorption spectra of the leaf pigments of the plants from all three groups (Figure 4) indicated that there is a difference among the plant groups. The “control” group seems to have higher absorption and the “cold” group drops to the lower values, while the “heat” group is accommodated somewhere in between. However, the differences cannot be considered as statistically significant because the standard errors seem to cover the whole area between the graphs.

### 2.3. DDC Expression

**Figure 5:**
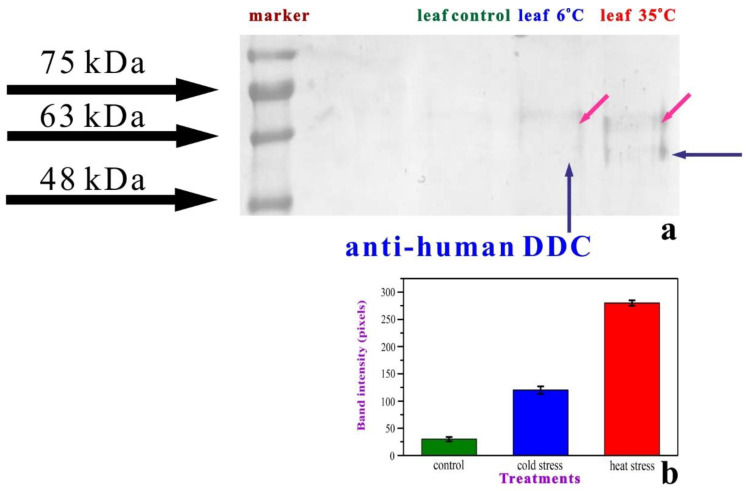
The immunodetection of DDC (**a**) and bar graph for the quantification of the band intensity (**b**) in the leaf samples of control and exposed plants (small blue arrows). The upper band (pink arrows) probably indicates a weak expression of another transcript of the DDC, with an approximately similar molecular weight.

Interesting results were also acquired through the immunodetection of DDC in the leaf samples of control and stressed plants (Figure 5). In the plants of the “control” group (**L1**), no expression of the enzyme can be detected. However, the levels of DDC protein expression rise remarkably in the leaves of the “cold” group (**L2**) and appear significantly increased in the leaves of the “heated” plants (**L3**). This fact is also clearly demonstrated in the band intensity graph (Figure 5, lower).

The obtained data indicate the presence of two DDC immunoreactive signals in the tested samples. The presence of the higher MW DDC immunoreactive band could represent a yet unknown DDC isoform or post-translational modification. The upper band, in all specimens (Figure 5, **hot pink** arrows), probably indicates a weak expression of another transcript of the DDC, with an approximately similar molecular weight. 

**Figure 6:**
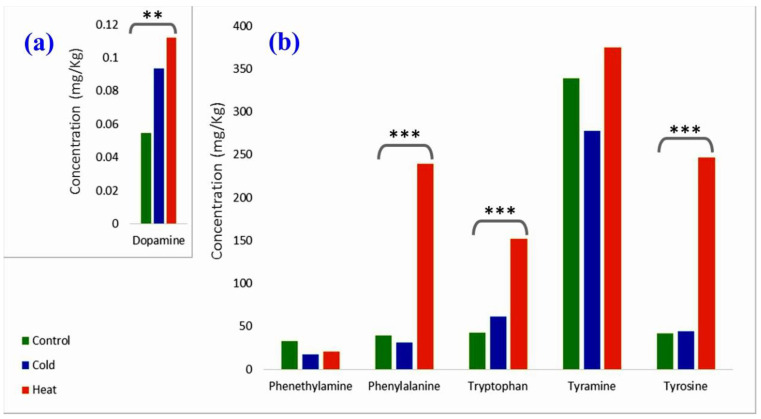
Bar chart of the average concentrations of the quantified amines in *P. lentiscus* plants grown under different temperature conditions. Control group is presented with green bars (control), low temperature plants with blue bars (cold), and high temperature plants with red bars (heat). (**a**) Representation of the low-concentration amines (dopamine). (**b**) Representation of high-concentration amines/amino acids (phenylethylamine, phenylalanine, tyrosine, tryptophan, tyramine). Results are expressed in mg/kg of dried leaves. ** (*p* ≤ 0.05) and *** (*p* ≤ 0.001).

Additionally, in the current study, specific stress-related amines were quantified in the three plant groups. Amines were discriminated in two different levels based on their endogenous content in control plants: low- and high-level amines. Generally, for stress groups, the application of high temperature seems to have a significant effect on amine production in comparison to low temperature. In more detail, regarding low-level molecules, dopamine showed the lowest concentration in all treatments, while tryptamine and serotonin (both derive from tryptophan decarboxylation) were not detected, neither in the control nor in the stressed plants. For the high-level molecules, phenylethylamine and tryptophan presented the lowest values for plants exposed to high temperatures; phenylalanine and tyrosine possessed medium values and tyramine presented the highest values. The quantitation of the results for the detected amines for all plant treatments is illustrated in Figure 6 and Table S1.

According to Figure 6, most of the quantified molecules demonstrated an increasing trend in their concentration when plants were treated under stressing temperatures in comparison to control plants. Generally, high temperature presented a significantly stronger impact on amine production than did low temperatures. Phenylethylamine and tyramine were the only amines that did not follow this trend (Figure 6b). The control group of phenylethylamine demonstrated higher concentrations in comparison to both stressed groups, and the control group of tyramine demonstrated higher concentrations in comparison to cold plants. To evaluate the significance of the differences between stressed plants and the control group, results were processed with ANOVA to determine the *p*-value for each metabolite (Tables S2–S4). 

Based on ANOVA calculations, high and low temperatures reveal a significant effect on dopamine concentration (Figure 6a). Plants increase their dopamine levels under stressing temperatures—more specifically, application of high temperatures revealed a stronger effect on dopamine production compared to low temperatures.

Regarding the quantitation of compounds detected in high concentrations, the application of high temperature revealed statistically significant effects on the amine biosynthesis in comparison to the control group, while low temperature has no significant effect (Tables S4 and S5). Tyramine was the only exception in this trend, as stress conditions had no effect on its concentration.

## 3. Discussion

Optical microscopy revealed that the control leaves significantly differ when compared to those of the stressed plants (Figure 1a–c_1_). The leaves of the control plants seem to follow the common habit of Mediterranean plant life. They accumulate phenolics within the vacuole of most of the mesophyll cells. Tissue arrangement seems normal. The palisade parenchyma occupies about half of the mesophyll, while the spongy parenchyma, which hardly accumulates osmiophilic metabolites, exhibits distinct intercellular spaces. The rather flat epidermal cells also appear free of osmiophilic inclusions, while the resin ducts of the leaves retain their normal structure [21]. The above-described tissue arrangement seems to be somehow disturbed when it comes to leaves subjected to either heat or cold stress [1]. The epidermal cells remain flat but accumulate granular, osmophilic material. In cold-stressed plants, this material appears denser while the palisade cells accumulate phenolics. The spongy parenchyma (abaxial) of the cold-stressed leaves exhibits a very compact structure in contrast to the same parenchyma of the control leaves, while its cells are fully impregnated with condensed phenolics. In all three leaf types, idioblasts containing druses seem to be common, while the resin ducts do not seem to be disturbed. Resuming the results of the structural observations, we can make it clear that the leaves of both the heat and cold-stressed plants turn seriously xeromorphic [40]. Although it has been demonstrated that Mediterranean evergreen sclerophylls evolved through a constant struggle against the hot and dry Mediterranean summer [8], the brief, unpredictable chilling temperatures during winter are suspected to be equally bothering [41]. 

After investigating the final yield for the three plant groups (Figure 2), we may suggest that the evergreen sclerophylls, while they “try to defend” themselves from the stressful Mediterranean summer, probably “underestimate” the brief wintertime period when the below-zero temperatures occur [41]. This is probably the reason why *P. lentiscus* suffers a significant biomass reduction when grown at low temperatures [42]. Moreover, the distribution of this species along Greece’ s mainland was reported to be strongly affected by low temperatures [43]. Control and heat-stressed plants exhibit only minor differences in their development, while the cold-stressed plants are significantly retarded. It seems that the summer stress is manipulable by these plants, while the brief yet severe chilling conditions during the winter months are still a “hard-nut-to-crack” for Mediterranean plant life.

On the other hand, individuals exposed to high temperature appear to experience severe oxidative stress, significantly higher than that of their cold-stressed counterparts and incomparably increased when considered along with the hardly existing oxidative stress of the normal plants (Figure 3) [44]. This is due to the impact the high temperatures have on the photosynthetic apparatus [45]. The precious crossroad, canalizing plant metabolism to the secondary product biosynthesis, seems to start right here [46].

Concerning the absorbance of the leaf photosynthetic pigments, although the differences can be considered as statistically non-significant, we observe that heat-stressed plants do present a decrease of the pigments, but when it comes to the cold-stressed plants, the reduction seems more severe, at least at the short wavelength side of the spectrum. Because the absorbance is given in A.U. per fresh weight, its reduction in the stressed leaves is probably the result of ROS build-up [47], the mid-winter chronic photoinhibition, and the leaf chlorophyll contents [48].

The interesting results of this investigation will emerge when starting to evaluate how each one of the individual parameters is involved in the overall manipulation of the cold or heat escape strategies of the plant.

Because the heat stress emerges as a serious challenge under projected global warming, the frequency and intensity of the heat waves will become more severe [49]. By extending our knowledge regarding the ecosystem reactions and the strategies that the plants have developed in order to escape the heat strain, new methods may be revealed for improving crop resistance to heat events. This is a paramount scientific challenge that may affect the future of the globe [49,50,51]. 

The production of reactive oxygen species (ROS), generally attributed to heat stress in plants, causes damage on a wide array of cellular components through disrupted membrane stability or reduced energy metabolism [52,53]. Other types of stress (i.e., LF-EM radiation, GSM radiation) [12,54,55] also induce ROS production, while the data of the current investigation revealed that ROS are also produced after a cold stress, although in inferior quantities. However, the increase in ROS within the plant cell imposes the prompt release of antioxidant molecules to scavenge the detrimental effect of these oxidative factors [7,19]. This is true for many of the Mediterranean plants investigated [21].

**Figure 7:**
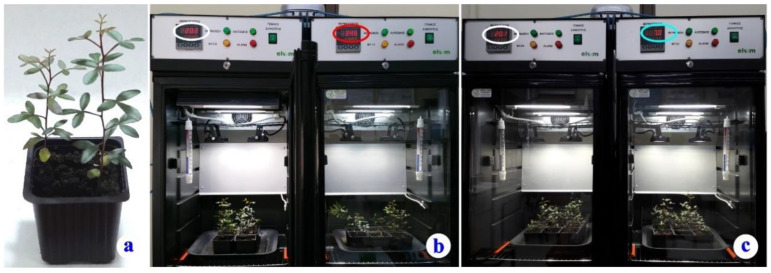
(**a**). One of the pots with two *P. lentiscus* plantlets, at the beginning of the experiment. (**b**) Two of the culture chambers during the heat stress. The control chamber, at the left, and the heat-stress chamber, at the right. Note the temperature reading (34.5 °C) at the moment of the snapshot. (**c**) Two of the culture chambers during the cold-stress experiment. Note the temperature reading (7.0 °C) at the right chamber.

After carefully evaluating the current data, we realized that 10-day severe heat stress in *P. lentiscus* induces the biosynthesis of secondary metabolites (Figure 1), slightly reduces biomass production (Figure 2), boosts ROS production by more than 400%, compared to the control plants (Figure 3), lowers the photosynthetic pigments’ absorbance to a non-significant extent (Figure 4), and triggers DDC expression over 1000%, compared to the control plants. In the same context, while DDC expression increases to extreme values after the heat stress (Figure 5), the heat-stressed plants adjust their amine content and overproduce specific stress-related biogenic amines (Figure 7). 

**Figure 8:**
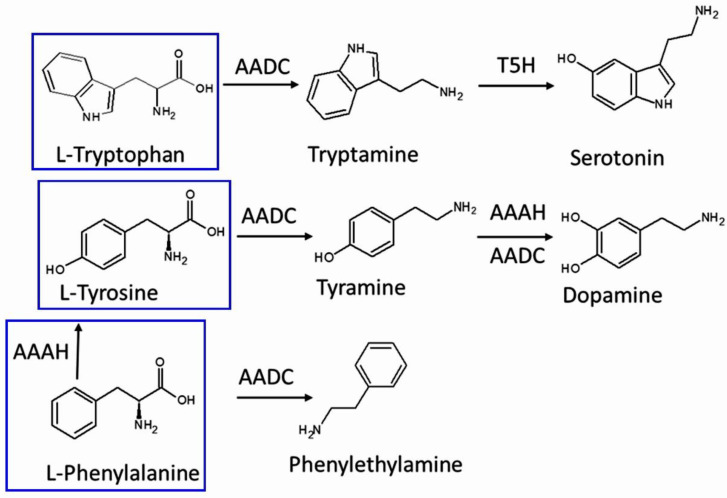
Chemical structures and biosynthetic formation of the selected biogenic amines/amino acids (tryptophan, tryptamine, serotonin, tyrosine, tyramine, dopamine, phenylethylamine, and phenylalanine). AADC: Aromatic amino acid decarboxylase (DDC); T5H: Tryptamine-5-hydroxylase; AAAH: Aromatic amino acid hydrolase.

Impressively, heat stress plants synthesize large quantities of three out of the eight amines selected for this investigation: phenylalanine, tryptophan, and tyrosine (Figure 6b and Figure 8). Among the less abundant molecules investigated, dopamine presented the highest average concentrations (Figure 6a and Figure 8). Tryptamine and serotonin were not detected at all, in all samples. This happens either because these heterocyclic amines are very sensitive, thus having low half-time life, or because they are rapidly bio-transformed to other chemical structures, such as melatonin, which is a downstream product of serotonin biosynthesis [56]. Previous research revealed that heat-stress induces the elevation of enzyme activities (serotonin N-acetyltransferase and hydroxyindole-O-methyltransferase) that regulate the final formation of melatonin from serotonin in rice seedlings [56]. Melatonin is considered a powerful antioxidant agent if compared to serotonin and tryptamine [57]. This observation, in combination with the results of Figure 3, illustrating the increased ROS production in cold and heat-stressed plants, reinforces the hypothesis of the bio-transformations of some compounds to more potent antioxidant structures.

The biosynthesis of the three high-concentration amines in cold-stressed plants seems to be low. The same is true for their control counterparts (Figure 6), although tryptophan is also frequently reported at higher concentrations under drought stress [58]. What seems probable, in general, is that the presence of the three amino acids—phenylalanine, tryptophan and tyrosine—triggers or at least “betokens” the mobilization of the secondary metabolite biosynthesis [59]. It is interesting that phenylalanine and tyrosine, which appeared in medium values, are intermediate metabolites for the production of other studied molecules: tyrosine for dopamine and tyramine as well as phenylalanine for phenylethylamine (Figure 7). Based on the literature, their production, coming through the shikimate pathway, is enhanced under heat stress conditions because they are the precursors of the numerous metabolites involved in stress defense [60]. Tryptophan is a precursor for most indole compounds, including two plant hormones, auxin and melatonin, which promote plant tolerance to various abiotic strains [61]. Regarding phenylalanine, the precursor of tyrosine, previous research revealed that its biosynthetic pathway is also enhanced under low temperatures [62]. In addition, phenylalanine is the precursor of the phenylpropanoid biosynthesis pathway [63], which generates numerous phenolic compounds whose accumulation in plants has also been associated with heat stress conditions [21]. In the meantime, the considerable increase in DDC expression (Figure 5) may promptly convert L-DOPA to dopamine [28] so that the consequent dopamine output (Figure 6A) keeps level with the urgent need for ROS scavengers and the pigments recur to their normal absorption; thus, the cells reset their metabolism [21]. Dopamine, a catecholamine possessing a 3,4-dihydroxy-substituted phenyl ring, has the necessary structure for a potent antioxidant [64] and acts as a quick ROS scavenger [65]. Dopamine is considered a significant neurotransmitter in animals and humans, being the precursor in the biosynthesis of other related catecholamines such as norepinephrine and epinephrine, which are also known for their hormonal action in mammals [66]. Recent studies revealed that dopamine enhances tolerance to drought and salt stress, as well as to nutrient deficiency in plants [67] and particularly low and high temperatures have been found to increase the activity of the important enzymes involved in catecholamines’ biosynthesis, such as tyrosine hydroxylase and DDC, in potatoes [68]. Dopamine has a short half-life in humans or other mammals [69]. It varies from one minute to 60 ms [70,71]. The half-life of dopamine for the plant cells has not been specified, yet it has been demonstrated that dopamine is soon converted to hydroxytyrosol (HT, IUPAC name: 4-(2-Hydroxyethyl)-1,2-benzenediol)). We may suggest that this is the reason why the significant rise of DDC in the stressed leaves is also followed by the increase in HT concentration. L-DOPA and L-tyrosine are both metabolized through the same pathway [21].

## 4. Material and Methods

### 4.1. Plant Material, Experimental Set-Up and Culture Conditions 

Seeds of *Pistacia lentiscus* var. *chia*, obtained from Templiner Krautergarten (Storkower Dorfstr. 53, 17268, Templin, Germany), underwent acid scarification for breaking their dormancy. They were treated initially with concentrated (98%) sulfuric acid (pro-analysi, MERCK) for 5 min [72] and then with 37% hydrochloric acid (pro-analysi, MERCK), for 5 min, at room temperature. This type of scarification was used because it simulates the effect of the acidic environment in a bird’s gastrointestinal track and seems to justify an old Mediterranean adage assuring that the mastic tree seeds cannot germinate unless they are consumed by a thrush. After the acidic treatment, the seeds were washed thoroughly to remove any residues. Germination tests were performed in glass Petri dishes (9 cm diameter) on 2 sheets of Whatman No. 1 filter paper moistened with de-ionized water [73]. Seed germination took place in a chamber at 20 ± 2 °C, humidity 70%, light/dark cycle of 16/8 h, and illumination supplied by cool-white fluorescent tungsten tubes (Osram, Germany) at 110 μmol/m^2^s Photosynthetically Active Radiation (PAR).

The seeds germinated after about 30 days. Seedlings were then sown in 10 × 10 × 11 cm, 1.10 L pots filled with wet Potgrond P medium (Klasmann–Deilmann), at pH 6.0. The composition of Potgrond P, the plant-growing medium, is: frozen through black peat; organic matter 90–95% (*w*/*w*); humidity: 60–70% (*w*/*w*); conductivity: 40 mS/m (+/−25%); pH (H_2_O): 5.5–6.5; (NPK Fertilizer 14:10:18): 1.5 kg/m^3^. Two seedlings were planted in each pot. The pots, each one with two young *P. lentiscus* plants (Figure 7a), were placed on trays. Nine pots (18 plants/treatment) were accommodated on each tray (total 54 plants). Because *P. lentiscus* is a slow-growing plant, the plantlets were left for development in the plant growth culture chambers for three months (from 15 December 2020 until 20 February 2021). 

The plant growth culture chambers had a built–in illumination source (two 1000 lumen, 10 W LED at 4000 K, assisted by two Osram basic, T5 Short, L 6 W/640 miniature tubes, length 212 mm, diameter 16 mm, 4000 K). Total illuminance at the surface of the pots, directly measured with a MASTECH MS6610 portable luxmeter (Mastech Group Limited-Taipei), was 201.4 umol/s/m^2^ to 204.8 umol/s/m^2^. The light/dark cycle of the chambers was adjusted to 16/8 h [54,55]. Air was supplied, in each of the chambers, through a HAILEA ACO-9160 (China), at an output of 4 L/min, for each chamber. 

After three months, the trays with the pots were used as follows: One tray with 9 pots (total 18 plants) was used as a control. Another tray (9 pots, 18 plants) was exposed to cold stress, and one more tray (9 pots, 18 plants) was used for the heat stress (Figure 7b,c).

The *P. lentiscus* plants were exposed to heat and cold stress according to the following set-up: The “control” group was cultured at 20 ± 0.5 °C, a moderate temperature common during the Mediterranean spring, considered as non-stressing for these plants (Figure 7b,c, chamber at left) [41]. Heat stress was applied by adjusting the culture temperature in one of the otherwise identically setup chambers, at 35 ± 0.5 °C (Figure 7b, chamber at right), while the cold stress was simulated in another chamber with a temperature of 7 ± 0.5 °C (Figure 7c, chamber at right). Relative humidity was measured using a Xiaomi Monitor 2–Mi ^TM^ (NUN4126GL), and was 60–65% for the heat stress chamber and 45–50% for the cold stress chamber. Air temperatures higher than 35 °C, although common during the day in a Mediterranean type ecosystem, normally do not remain constant; neither last for a long time while, during the three winter months, the lowest average monthly temperatures, around 8 °C, occur (https://en.climate-data.org/europe/greece/athens/athens-7/#temperature-graph (accessed on 25 October 2022)). The temperature strain lasted for 10 days. The trays received 90° clockwise rotation, daily, in both chambers, in order to eliminate any possible effect of unequal light or temperature dispersion. 

By the end of the stress period, the pots were taken out from the chambers, and all the plants were washed rapidly for the removal of any remnants of the culture medium. The three groups of plants were subjected to the following, identical treatment protocols, which will be described in detail later in this section: (1) small pieces from leaves of the same age were fixed for microscopy; (2) a small quantity of leaf material was used for (a) ROS estimation through lipid peroxidation (malondialdehyde-MDA), (b) determination of the photosynthetic pigments’ absorption, (c) investigation of the DDC expression; (3) the rest, which was the majority of the plant material, was immediately stored at −80 °C to preserve the aminic and/or amino acid content from any chemical degradation and/or biotransformation and used for the UPLC-tripleTOF MS analysis.

### 4.2. Plant Anatomy and Microscopy

For the anatomical/microscopical investigation, five plants were used, at random, and a composite leaf was detached from each one of them. Small pieces (1 × 1 mm) from an area in the middle of the leaf blades and adjacent to the central vein were excised. All these pieces together were fixed in phosphate buffered 3% glutaraldehyde (pH 6.8) at 0 °C for 2 h and post fixed in 1% osmium tetroxide in phosphate buffer for 2 h at 0 °C. The protocols for double fixation, embedding, sectioning, and light microscope observations are cited in detail by Christodoulakis et al. [74].

### 4.3. Photosynthetic Pigments Protocol

Fresh leaves from all treatments were submerged in 80% acetone for 48 h. The spectral absorbance of the photosynthetic pigments was measured using a VWR1200 © spectrophotometer and normalized per mass, according to the protocol described by Gechev et al. [19,75].

### 4.4. DDC Expression and Quantification

Protein extraction, determination of total protein concentration, SDS-polyacrylamide gel electrophoresis (PAGE), and immunoblotting were performed as cited in detail by Stefi et al. [20,21].

The specific anti-DDC antibody (against the last 22 C-terminal amino acids of human DDC) was raised in the laboratory of Dr D. Vassilacopoulou (Section of Biochemistry and Molecular Biology, Department of Biology, Faculty of Sciences, National and Kapodistrian University of Athens, Athens, Hellas (Greece)). The secondary antibody (MFCD00162782) Anti-Rabbit IgG conjugated with Alkaline Phosphatase, was purchased from Sigma-Aldrich™, Milan, Italy. The chromogenic alkaline-phosphate detection method was performed on the nitrocellulose membrane. Nitro Blue Tetrazolium/5-Bromo-4-Chloro-3-Indolyl Phosphate (NBT/BCIP Merck KGaA, Darmstadt, Germany) substrate was used as the detection method.

Software “Image Pro Plus” v.10.0 was employed for the quantification of bands of immunodetection. The intensity of each band was measured 5 times in order to have the statistically needed repetition for the standard error of the mean, and data (in pixels) were illustrated in bar graphs, using the software “OriginLab Pro” v.9.0.

### 4.5. Total Reactive Oxygen Species (ROS) Estimation

ROS levels were estimated via lipid peroxidation using the MDA method; 50 mg of frozen plant material was homogenized and mixed with 1 mL 0.25% thiobarbituric acid (TBA) dissolved in 10% trichloroacetic acid (TCA) solution, so as to extract the MDA. The extract was collected and heated at 85 C for 30 min. The mixture was centrifuged at 13,000× *g* for 10 min, and the supernatant was transferred to a cuvette for photometry. The absorbance was read both in 532 nm (MDA-TBA peak) and 600 nm (non-specific absorbance). The MDA concentration was estimated using formulas described by Gechev et al. [75].

Total ROS were expressed as nmoles MDA/g of leaf tissue.

### 4.6. Statistical Analysis

All numerical data being subject to statistical analysis were processed using the SPSS v.21.0 software (SPSS Inc., Chicago, IL, USA). The analysis was performed by employing paired t-tests comparing control samples to each one of the exposed. Data were previously checked for their normality. Data were expressed as mean ± standard error of the mean. The differences were considered to be statistically significant when *p* < 0.05 (*). *N*: number of biological samples.

### 4.7. Selection of Biogenic Amines/Amino Acids for UPLC-Triple TOF MS Quantitation

Biogenic amines/amino acids are considered as important regulators of plant growth. Moreover, they display significant pharmacological effects on humans and are also important precursors of hormones and components of coenzymes [76]. Further investigations demonstrated that biogenic amines serve as vital neurotransmitters in the human central nervous system [76]. The formation of biogenic amines in all living organisms comes from the decarboxylation of amino acids, by the removal of the *α*-carboxyl group to give the corresponding amine [77]. 

In the current study, the selection of the biogenic amines/amino acids under investigation was based on the biosynthetic pathways involved and their role in DDC expression [76]. Specifically, eight aromatic and heterocyclic molecules were selected for the investigation: tyramine, tyrosine, tryptamine, serotonin, tryptophan, phenylethylamine, phenylalanine, and dopamine. Based on the existing literature, these amines are precursors or intermediate metabolites of compounds associated with plant exposure to stress conditions or are related to DDC activity. The chemical structure of these amines, selected for quantitation, is illustrated in Figure 8.

### 4.8. Method Development and Assessment—Quantitation of Biogenic Amines/Amino Acids in Pistacia Lentiscus Leaves via UPLC-TripleTOF MS

For the method development and the evaluation of the extraction recoveries of the selected molecules, calibration samples from wild-growing *P. lentiscus* young plants were used. These calibration specimens were found to be positive in some of the amines/amino acids due to their endogenous production. The latter led to the incorporation of the standard addition method for the evaluation/check of the developed methodology. As quality control samples, three replicates of the calibration *P. lentiscus* samples were used for the validation of the method and two triplicates of spiked samples (control, heat, and cold) at the validation’s levels were used to ensure analysis reliability. The optimal extraction protocols were applied in triplicate to verify the method’s accuracy (recoveries within batch 66–124%, between batch 37–103%, repeatability (RSD%_W_ 3–15%) and reproducibility (RSD%_B_ 10–19%)) (Table S5). 

After the extraction of *P. lentiscus* leaves, the three different extracts coming from the control group, the group grown at low temperatures (cold), and the group grown at high temperatures (heat) were analyzed and quantified for the determination of their acute concentration in the selected molecules. The quantification of control samples indicated the values of the amines’ endogenous production in the typical growing conditions of the Mediterranean spring and allowed the conduction of comparisons between samples and statistical evaluation thereof. 

Quantitation results were grouped in two different categories: low- and high-concentration amines. As already reported, the expected levels of some amines in the control plants fall into two scales of quantitation, i.e., low-concentration levels (dopamine, serotonin, and tryptamine) and high-concentration levels (phenylethylamine, phenylalanine, tyrosine, tryptophan, and tyramine). 

### 4.9. Extraction and Sample Preparation for the Analytical Part

Prior to extraction and analysis, the leaves of *P. lentiscus* were separated from the roots and lyophilized for 48 h. The dried samples were pulverized to achieve sample uniformity. Due to the high differences in the concentration levels of the various amines or amino acids, two extraction protocols were developed, incorporating liquid–liquid extraction (LLE) to ensure a high recovery of amines or amino acids. For those of such compounds found normally in high concentrations in plants, i.e., phenethylamine, phenylalanine, tyrosine, tryptophan, and tyramine, 10 mg of leaves from the three different treatments (control, cold, and heat) were mixed with 1 mL of water (H_2_O) acidified with 5% trifluroacetic acid (TFA) and vortexed for 2 min. Then samples were sonicated for 1 h with temperature control set at <20 °C. Finally, the samples were centrifuged at 8000× *g* for 8 min to separate the aqueous extract, which was directly analyzed using UPLC-triple-TOF MS. For the low-concentration amines, i.e., serotonin, dopamine, and tryptamine, 10 mg of leaves from the different treatments were extracted with 1 mL of methanol (MeOH) acidified with 2% acetic acid (AA) employing the same procedure as in the previous protocol, with the only difference of the addition of an evaporation step before analysis and sample reconstitution in 200 μL H_2_O acidified with 0.2% formic acid (FA).

### 4.10. Quantitative Analysis of Biogenic Amines and Amino Acids via UPLC-TripleTOF MS

For the quantitative analysis, an UPLC system (Acquity H class, Waters Corporation, U.S.A.) coupled to a hybrid quadrupole-TOF LC–MS/MS mass spectrometer (Old Connecticut Path Framingham, MA 01701 USA.) equipped with a DuoSpray ion source was incorporated. For the chromatographic elution, a gradient system consisting of H_2_O with 0.01% FA and 10 mmol ammonium formate (A) and MeOH with 0.01 % FA and 10 mmol ammonium formate (B) was used. For the chromatographic separation, a Supelco C18 (150 × 4.6 mm, 5.0 μm) column was used, and the column temperature was set at 35 °C. The analysis was performed with a linear gradient method starting with 10% of B, reaching 75% in 15 min, and finally with 5 min of equilibration time. The flow rate was 0.5 mL/min, and the injection volume was set at 10 μL. Spectra were obtained in positive ion mode, and for the spectrometric analysis capillary voltage was set at 4500 V; source temperature was adjusted to 500 °C, gas1 and gas2 to 45 psi, and curtain gas to 30 psi. Data were acquired with the SWATH method and rolling collision energy. The mass spectrometer parameters of the employed method are presented in detail in Table S6 in the Supplementary Material. System control was performed with Analyst TF 1.7.1 and the data process with Sciex OS. For compound identification, three independent parameters were taken into consideration: retention time (RT), precursor ion accurate mass, and library match sample’s product ion MS/MS spectra in comparison to the reference spectra from our in-house spectrum library.

### 4.11. Statistical Analysis of Quantitation Results

All extractions and analyses were performed in triplicate, and the relative standard deviation (RSD %) was estimated for each biological or analytical triplicate, with acceptable measurements being those with RSD ≤ 20%. Statistical significance of quantitation results was evaluated using one-way analysis of variance (ANOVA) of MS Excel. Results denoted the 1-tailed distribution *p*-values for each metabolite. *p*-values ≤ 0.05 were characterized as significant, indicated with two asterisks in graphs, and *p*-values ≤ 0.001 with three asterisks in graphs.

## 5. Conclusions

*P. lentiscus* plants exposed to heat or cold stress modify the biosynthesis of their secondary metabolites accordingly, adjust their biomass production, boost ROS, and trigger intense DDC expression. By shifting their secondary metabolic pathways, stressed plants can synthesize certain metabolites, some of them highly exploitable, according to the stress they have experienced.

## Data Availability

Not applicable.

## Supplementary Materials

The following supporting information can be downloaded at: https://www.mdpi.com/article/10.3390/plants11233290/s1.
