# Supplementary material for: Heat and Cold-Stressed Individuals of Pistacia lentiscus (Mastic Tree) Do Modify Their Secreting Profile
Source: Plants (Basel). 2022 Nov 29;11(23):3290. doi: 10.3390/plants11233290 (PMC9736404; doi:10.3390/plants11233290)
Supplement: Supplementary file 1 [file plants-11-03290-s001.zip › plants-2038564-supplementary.pdf]

Table S1: Average concentrations of the biogenic amines/amino acids in samples. Results are expressed as mg/kg of dried leaves

Low concentration metabolites (mg/kg)			
**Compound**	**Control**	**Low Temperature**	**High Temperature**
**Dopamine**	0.055	0.093	0.112
**Serotonin**	ND	ND	ND
**Tryptamine**			
**High concentration metabolites (mg/kg)**			
**Phenethylamine**	32.8	17.4	20.9
**Phenylalanine**	39.4	30.8	239.3
**Tyrosine**	41.7	44.2	246.4
**Tryptophan**	42.2	64.0	151.6
**Tyramine**	339.2	278.1	375.2

ND = Not Detected

Table S2: Representation of p-values for all samples

All treatments				
**Dopamine**				
**Samples**	**rep1**	**rep2**	**rep3**	**p-value**
Control	0.045		0.064	0.042089
cold	0.079	0.108	0.093	
heat	0.134	0.09	0.112	
**Phenethylamine**				
Control	31.079	39.366	28.041	0.017254
cold		16.815	18.053	
heat	22.871	22.148	17.709	
**Phenylalanine**				
Control	32.374	40.493	45.262	1.07E-05
cold	28.694	35.112	28.541	
heat	265.094	206.791	246.08	

Tryptophan				
Control	45.664	38.676		0.002295
cold	38.676	79.86	65.058	
heat	137.239	141.779	175.695	
**Tyramine**				
Control	352	341.529	324.062	0.217555
cold	208.85	323.595	301.838	
heat	279.424	412.337	433.767	
**Tyrosine**				
Control	41.002	34.444	49.658	0.00011
cold		39.714	48.729	
heat	228.691	228.259	282.206	

Table S3: Representation of p-values for control samples and samples developed under low temperatures

Control vs cold				
**Dopamine**				
**Samples**	**rep1**	**rep2**	**rep3**	**p-value**
Control	0.045		0.064	0.057435
cold	0.079	0.108	0.093	
**Phenethylamine**				
Control	31.079	39.366	28.041	0.039366
cold		16.815	18.053	

Phenylalanine				
Control	32.374	40.493	45.262	0.118822
cold	28.694	35.112	28.541	
**Tryptophan**				
Control	45.664	38.676		0.313952
cold	38.676	79.86	65.058	
**Tyramine**				
Control	352	341.529	324.062	0.165956
cold	208.85	323.595	301.838	
**Tyrosine**				
Control	41.002	34.444	49.658	0.728252
cold		39.714	48.729	

Table S4: Representation of p-values for control samples and samples developed under high temperatures

Control vs heat				
**Dopamine**				
**Samples**	**rep1**	**rep2**	**rep3**	**p-value**
Control	0.045		0.064	0.048612
Heat	0.134	0.09	0.112	
**Phenethylamine**				
Control	31.079	39.366	28.041	0.033565
Heat	22.871	22.148	17.709	

Phenylalanine				
Control	32.374	40.493	45.262	0.00034
Heat	265.094	206.791	246.08	
**Tryptophan**				
Control	45.664	38.676		0.006264
Heat	137.239	141.779	175.695	
**Tyramine**				
Control	352	341.529	324.062	0.503155
Heat	279.424	412.337	433.767	
**Tyrosine**				
Control	41.002	34.444	49.658	0.000375
Heat	228.691	228.259	282.206	

Table S5: Full data of the method assessment applied in triplicate using the optimal extraction protocols.

Compound	Matrix Concentration (mg/Kg)	Spiked Level (mg/Kg)	Recovery (%)	RSD%	Linearity Range mg/Kg	R^2	Extraction solvent
**Dopamine**	0	0.500	66%	7%	0.005-0.100	0.99753	MeOH 2% AC
**Serotonin**	0	0.500	74%	9%	0.005-0.200	0.99326	
**Tryptamine**	0	0.500	60%	7%	0.005-0.100	0.9897	
**Phenylalanine**	224.7	100.0	97%	9%	0.005-0.500	0.99862	H_2O 5% TFA
**Tryptophan**	116.3	100.0	68%	12%	0.025-0.500	0.99619	
**Tyrosine**	279.6	100.0	107%	10%	0.01-0.500	0.99858	
**Phenethylamine**	10.2	10.0	124%	3%	0.00-0.200	0.99306	
**Tyramine**	17.3	20.0	99%	15%	0.005-0.100	0.98158	
**Hydroxytyrosol**	0.200	0.500	60%	6%	0.01-0.500	0.99481	MeOH 2% AC

Table S6: Mass spectrometry parameters employed for the quantitation of amines via UPLC-Triple-Tof MS

Compound Values	
**DP**	30
**CE Full Scan**	10
**CE SWATH**	Rolling
**CES**	15
**TOF MS AT**	0,25 sec
**TOF MS Masses**	80 - 570 m/z
**SWATH experiment AT**	0.065 sec
**SWATH Masses**	80- 600 m/z
**Product Masses**	50 - 600 m/z
**SWATH windows**	12
**Period Cycle time**	1.08 sec
